# The primate claustrum as a hub for precision weighting of prediction errors across the cortical hierarchy

**DOI:** 10.1371/journal.pbio.3003906

**Published:** 2026-07-16

**Authors:** Yujie Hou, Alessandra Pizzuti, Kenneth Knoblauch, Julien Vezoli, Rainer Goebel, Martin Vinck, Henry Kennedy

**Affiliations:** 1 Univ Lyon, Université Claude Bernard Lyon 1, INSERM, Stem Cell and Brain Research Institute U1208, Bron, France; 2 Department of Cognitive Neuroscience, Maastricht University, Maastricht, Netherlands; 3 Ernst Strüngmann Institute (ESI) for Neuroscience in Cooperation with Max Planck Society, Frankfurt am Main, Germany; 4 Donders Centre for Neuroscience, Department of Neurophysics, Radboud University Nijmegen, Nijmegen, Netherlands

## Abstract

Understanding how signals are exchanged across the cerebral cortex is a central challenge for theories of perception and cognition. Although crucial gaps exist in empirical knowledge, evidence is emerging in macaques for complementary functional roles of cortico-cortical and cortico-claustral-cortical loops in predictive coding and perceptual inference. This Essay puts forward a roadmap for investigating these cortical networks in the primate brain by integrating parallel neuroimaging investigation in humans with invasive electrophysiology and tracing experiments in macaques. It proposes that there are several anatomical features of the claustrum that promote signal integration, including precision-weighting across the primate cortical hierarchy, which merits further investigation.

## Abstract 2

Understanding how signals are exchanged across the cerebral cortex is a central challenge for theories of perception and cognition. This Essay puts forward a roadmap for investigating cortical networks in the primate brain, proposing that there are several anatomical features of the claustrum that may promote signal integration, which merits further investigation.

## Introduction

The cerebral cortex comprises constellations of sensory, motor, and associative areas linked by dense ascending and descending pathways across a hierarchical architecture. Understanding how signals are exchanged across this hierarchy is a central challenge for theories of perception and cognition that remains incompletely understood. In this Essay, we propose a roadmap for investigating these cortical networks in the primate brain.

The relative merits of generative and discriminative theories of perception are still debated, reflecting their importance for understanding human cognition. We first review theories of cortical function and situate discriminative and generative models (Box 1) with respect to the hierarchical organization of cortex. This provides the basis for evaluating predictive processing accounts of cognition in humans while highlighting the need for detailed circuit descriptions in non-human primates (NHPs). We use hierarchical predictive coding (hPC; Box 1) as a heuristic framework for organizing circuit questions but remain agnostic as to whether cortical hierarchies ultimately implement classical residual-error coding, feature-selective sensory amplification, precision-weighted (Box 1) hybrid schemes, or different solutions in different circuits. Recent work has emphasized that neural responses often described as ‘prediction errors’ (Box 1) may be similar at the response level while reflecting distinct underlying computations [1]. Our roadmap is therefore aimed not at presupposing a single canonical algorithm, but at identifying the circuits and signals required to discriminate among competing predictive architectures.

**Box 1. Glossary**

**Discriminative model**

A computational model that directly maps sensory inputs to outputs (e.g., object recognition) without explicitly modeling the underlying causes of the data.

**Generative model**

An internal probabilistic model that predicts sensory input by modeling the hidden causes of observations.

**Hierarchical predictive coding**

A framework proposing that predictions flow downward in a hierarchy while prediction errors flow upward to refine internal models of sensory causes.

**Precision weighting**

The process of scaling prediction errors by their estimated reliability, determining how strongly they influence belief updating.

**Prediction error**

The difference between predicted sensory input and the actual input received. Prediction errors are propagated up the cortical hierarchy to update higher-level predictions and improve the brain’s generative model.

**Precision**

A measure of the reliability or confidence assigned to prediction errors or sensory inputs, often associated with attentional modulation.

**Cortical hierarchy**

The layered organization of cortical areas where processing progresses from simple sensory representations to increasingly abstract cognitive representations.

**Bottom–up processing**

Information flow from lower sensory areas toward higher cortical areas, typically carrying sensory evidence or prediction errors.

**Top–down processing**

Signals sent from higher to lower cortical areas that convey predictions, contextual information, or attentional modulation.

**Inference**

The process of estimating hidden causes of sensory inputs based on current evidence and prior beliefs.

**Priors**

Beliefs or expectations about the world held before observing new sensory evidence.

**Tract tracing**

A set of experimental methods used to map neural connectivity by labeling pathways between brain regions. Tract tracing can identify the direction, weight, and targets of projections.

**Cell type**

A classification of neurons or other cells based on shared biological properties, such as gene expression profiles, morphology, connectivity, electrophysiological characteristics, or developmental origin. In modern neuroscience, cell types are increasingly defined using transcriptomic signatures, allowing fine-grained distinctions between neuronal populations involved in specific circuits or computations.

**Prediction**

A top–down signal generated by higher cortical levels representing expected sensory input.

**Active inference**

A theory proposing that the brain minimizes prediction errors by updating internal beliefs and by acting on the environment to make sensory inputs conform to predictions.

**Spatial transcriptomics**

A set of molecular techniques that measure gene expression (RNA transcripts) while preserving spatial information within tissue. Unlike traditional transcriptomics, which dissociates cells, spatial transcriptomics allows researchers to map where specific genes are expressed across brain regions, layers, or circuits, enabling the identification of spatially organized cell types and functional architectures.

**Oddball paradigm**

A type of mismatch paradigm in which rare deviants break a regular pattern; the brain’s response to those deviants is the mismatch signal, and mismatch sensitivity is how strongly the system distinguishes the oddball from the expected standard.

**Pulvinar nuclei**

A higher-order thalamic nuclei interconnected with multiple cortical areas in the dorsal and ventral streams and implicated in visual processing.

**Top–down transfer of contextual information**

The conveyance of contextual signals from higher-order areas that shape neuron responses in lower-order areas according to information outside their classical receptive fields.

**VIP→SOM disinhibitory motif**

A cortical circuit motif in which VIP interneurons inhibit SOM interneurons, reducing SOM-mediated inhibition of pyramidal-cell dendrites and thereby disinhibiting dendritic/synaptic integration.

In this Essay, we distinguish direct cortico–cortical pathways from cortico–claustral–cortical loops, which provide a major conduit for communication between areas (i.e., inter-areal). We propose that the claustrum, by virtue of its widespread cortical connectivity, implements precision control across the cortical hierarchy (Box 1). We also identify the critical gaps in knowledge that need to be addressed to move the field forward and outline how human and NHP studies need to be integrated to better explain hPC in the human brain.

## Theories and models of perception

**Fig 1:**
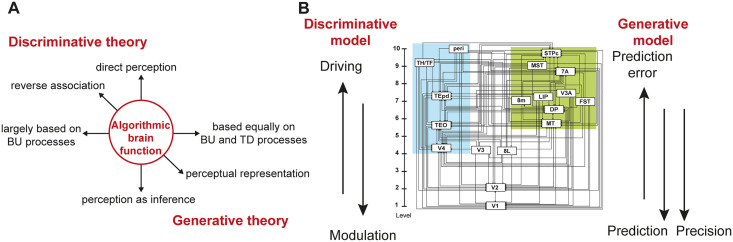
Discriminative and generative theories of perception. **A.** Rooted in two different traditions, discriminative (top) and generative (bottom) theories are characterized by symmetrical opposing features. Discriminative theories of perception envision perception emphasizing a bottom–up (BU) neural network that enables association, constructs discriminative receptive fields, and allows direct perception. Generative theories of perception are thought to emphasize equally BU and top–down (TD) networks, mental simulation, generative models, and inference. Hence, perception as inference views visual perception as a process that interrogates sensory evidence in relation to a generative model informed by prior knowledge of the world. While constructed on a symmetry of opposing features there is nevertheless an effort to integrate the two concepts. **B.** Discriminative and generative models of perception both accommodate the cortical hierarchical organization of Felleman and Van Essen [2]. Here, the cortical hierarchy is determinate and based on a quantitative measure of hierarchical distance [20]. In the discriminative model (left) BU signals are driving and the TD signals are modulatory. In the generative model (right) the BU signals convey prediction errors and the TD signals convey predictions and precision. Panel B adapted from [20].

An important clue to the function of the cortex in primates is its hierarchical organization. Generations of neuroanatomists have uncovered well-defined signatures of bottom–up (BU) and top–down (TD) connections (Box 1) linking hierarchically organized areas [2–4]. One view of brain function and perception is that the senses trigger sensory information flow and extraction in the BU pathways ascending the brain’s hierarchical levels [5]. Physiologists were quick to show that pathways ascending the hierarchy generate increasingly abstract representations of the world in higher areas, leading to the large range of receptive field properties observed at different levels of the hierarchy [6–13]. According to feedforward or discriminative theories of perception (Fig 1A), perception is enabled by the rapid analytical BU processing of features present in the sensory input generating increasingly complex representations of the sensorium (Fig 1B) [14]. However, the feedforward model largely ignores TD connections [15–19]. Recent anatomical studies have shown that TD feedback connections are twice as numerous as BU connections [20], and importantly, despite the ubiquity of collateralization of individual neuron projections in the cortex, individual inter-areal projection neurons virtually never possess collateral axons with TD and BU directed branches in macaques [21], although they do in mice [22]. In addition, TD pathways substantially contribute to perception [23] and can act on the earliest part of feedforward sensory response [24,25]. Furthermore, there is a long-standing recognition that prior knowledge of the world and contextual processing carried by TD pathways are necessary to make sense of incoming information. According to Helmholtz, virtually all perception involves some degree of internal reconstruction where perceptual inference (Box 1) enables the brain to build generative models that in turn support model-based perception [26] (Fig 1A). Perceptual inference is thought to require signals from the senses ascending the BU pathways to be integrated with internal expectations and priors (Box 1) according to an as-yet undetermined process [27,28] (Fig 1A, lower). This integration solicits a dynamic information exchange between distributed neuronal assemblies across brain areas, with distinct populations of BU and TD neurons that reside in and project to specific cortical laminae. Fig 1A suggests that discriminative theories of perception in the empiricist tradition and generative theories proposing perception via synthesis are composed of symmetrical opposite features (e.g., direct perception versus perception as inference, etc.).

Nearly all large-scale models of cortical function refer explicitly to cortical hierarchy. Numerous tract tracing (Box 1) experiments conducted between 1970 and 1990 showed that the laminar patterns of the origin and termination of inter-areal pathways stemming from the primary cortical areas to the association cortices allowed pathways to be categorized as feedforward (i.e., BU) and feedback (i.e., TD). Pairwise comparisons of these connectivity patterns made it possible to quantify the structural hierarchical organization of the cortex for model species including mouse and macaque [2,21,29,30]. These connectivity hierarchies correspond to hierarchies of transcription-defined cell types (Box 1) [22,31], as well as to electrophysiologically defined functional hierarchies [30,32].

Both discriminative and generative models of perception accommodate the cortical hierarchical organization with increases in the receptive field complexity and size while ascending the cortical hierarchy (Fig 1B). In discriminative models, BU signals are driving while the TD are conceived to be uniquely modulatory [33–35]. This contrasts with generative models, where the BU connections are also conceived to be driving and convey prediction errors, but the TD predictive connections (Box 1) are thought to be both driving (for the suppression of prediction errors via a subtractive effect) and modulatory (for the precision via synaptic gain control) [36,37]. While these models have distinct historical roots, a number of researchers argue both from computational resources and high-level vision perspectives that it might be necessary to consider hybrid models of direct perception versus perception as inference, where distinct computations are needed for different tasks [1,38–40]. In this Essay, we describe a comprehensive strategy to generate the data required to sufficiently constrain future models of perception whilst remaining agnostic to the possibility of hybrid models.

The past 20 years have seen a rapid development of predictive processing and active inference frameworks (Box 1), of which hPC is one influential formulation. hPC postulates that the hierarchical levels of the cortex are in the business of a dynamic updating of a statistical model of the causes of the sensory manifold [36,37,41–44]. According to hPC, the empirically observed TD signals of expectation or prediction [19] and modulatory precision-weighting of the ascending prediction error interact via an as yet undetermined circuit [45]. In the exchange of information between hierarchical levels, the (precision-weighted) prediction error has classically been conceived as the residue of an unresolved prediction and hypothesized to be a principal ascending signal in some hierarchical predictive-coding models, although recent work has questioned whether ascending sensory pathways encode residual differences per se, or instead convey sensory representations where the gain is modulated by TD predictions [1] (Box 2). While, ultimately, knowledge of the world is involved in TD prediction, in generative models, it is the multiple reiterations of prediction between individual areas across the hierarchical levels in conjunction with the aforementioned TD attention-like precision signals [46] that leads to a process of error minimization. Although the hypothesis that ascending pathways predominantly convey prediction errors has been questioned [47,48], it remains unresolved whether these signals encode residual differences, amplified sensory features, or more general surprise-related computations [1,48–51]. While non-invasive neuroimaging demonstrates TD predictions, crucial evidence at the neuronal and mesoscopic scales is still lacking [19]. This is particularly the case in primates, partly due to only very sparse knowledge concerning BU and TD pathway convergence and the lack of appropriate molecular tools [48].

**Box 2. The multiple roles of top–down influences**

Classical neurophysiological studies show bottom–up (BU) pathways ascending the hierarchy generate increasingly abstract representations of the world in higher areas, with each level processing sensory data within progressively larger windows of spatio-temporal integration [52–54]. By contrast, the functions of top–down (TD) pathways remain elusive and constitute a major impediment to understanding the brain. While theories of sensory processing have often assumed a singular function for TD influences, it stands to reason that TD pathways perform, in addition to the intensely studied spatial attention, not one but a host of complex operations. Hierarchical predictive coding (hPC) theory canonically recognizes two distinct TD operations: the comparison of internally generated predictions of sensory input with actual inputs, and a gain adjustment of inputs according to their estimated reliability (i.e., precision or degree of confidence in prior beliefs) [55]. In addition, feedback has been assumed to have a role in imagining sensory-like representations from concepts born of prior experience of visual objects, modifying the local representational detail consistent with global image interpretation and, finally, gating synaptic plasticity. Largely influenced by discriminative theories of perception, early investigations of hierarchical processing in the cerebral cortex made broad claims that BU pathways are essentially driving target areas, whereas TD pathways supposedly uniquely exert a modulatory influence [56]. This account of hierarchical processes is at odds with hPC, and there is ample counter evidence showing that BU and TD do not map in a straightforward fashion to driving versus modulatory processes [57–59]. Further, a strict dichotomy of the roles of BU and TD pathways would seem at odds with the multiple physiological roles that are imputed to TD control. For example, in the case of mental imagery, one expects TD to ‘write-in’ a pattern of neural activity in early visual areas [60] (i.e., operate a driving process), or alternatively selectively modulate the activity of specific spontaneously active cell assemblies. In the case of cancelling out self-generated sensory inputs through TD signaling (e.g., lack of sensation following self-tickling [61]), TD activity would appear to conform to the idea of being subtractive [37]. An alternative interpretation that illustrates the difficulty of specifying TD signals is that the lack of sensation in self-tickling is consequent to sensory attenuation, via suspending precision, in order to ignore the consequence of the action and so enable the action to be executed. In artificial BU (deep) neural networks trained with back propagation, the exclusive role of the nonbiological reverse error propagation is to improve the data transformations implemented by the BU projections via gradient descent. In the cortex, it is an open question as to how TD adjusts synaptic weights of BU projections. The multiple TD projections converging onto layer 1 provide a candidate pathway for such an adjustment of BU synaptic weight [62–65]. Here, TD projections impact the distal dendrites of pyramidal neurons, activating non-linear NMDA-receptor-dependent dendritic integration mechanisms in perceptual learning demonstrated in primary sensory cortex [66] and the control of learning in perirhinal cortex [67]. The adjustments of synaptic weights or efficacy on short time scales can be read as the precision weighting of prediction errors that are generally attributed to neuromodulatory effects on postsynaptic gain. These effects are typically associated with classical modulatory neurotransmitter systems, voltage-dependent NMDA receptor function, and subsequent control of synchronous gain [68–71], and are usually associated with interactions between pyramidal cells and fast-spiking inhibitory interneurons.

Theories of hPC have widespread psychological and philosophical implications for perception [50], cognition [72], language [73,74], episodic memory [75], and consciousness [76,77]. The corollary of this broad reach is that hPC as a theory of brain function brings together not only neuroanatomy, neurophysiology, and neurodevelopment, but also psychophysics, computational neuroscience, psychology, and philosophy of mind. In light of the widespread impact of these theories, especially related to high-level human cognition and, in order to better comprehend the importance of hPC for understanding the relation of the mind to the world [43], we argue that it is necessary to investigate prediction error minimization in parallel studies in humans and in NHP, where invasive techniques will provide insight on cellular mechanisms in the animal model, and the human imaging studies will reveal their relevance to perceptual mechanisms and their dysfunction in the human brain. Elsewhere, we have described the severe limitations of the mouse model for understanding primate cortical function [78–81]. However, projected experimental investigations in the more reductionist mouse model will provide additional crucial mechanistic insight that will complement, stimulate, and enrich the investigations that we propose here [82].

## Communication across the cortex

**Fig 2:**
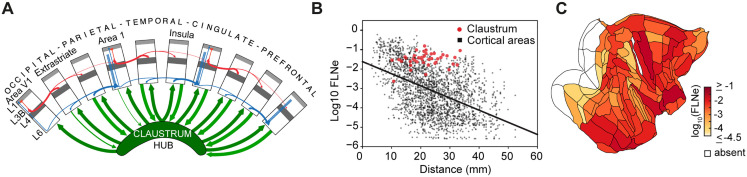
Cortico–claustral–cortical loops. **A.** A schematic of cortico–cortical connectivity, with cortico–claustral–cortical loops in green, direct bottom–up connections in red and top–down connections in blue. **B.** Weight–distance relationship with respect to the decline of the mean Log 10 FLNe index as a function of projection length (mm) for the full set of 2,342 cortico-cortical (black) and cortico-claustral connections in the G92x40 connectivity matrix [84]. Distances were estimated though the white matter (WM) from the injection site to the barycenter of the area containing labeled neurons, except for the early visual areas V1, V2, and V4, for which the injection sites were used. Claustrum distances were taken to be similar to the insula. Whereas the weight of cortico–cortical projections decreases exponential with distance, the claustral–cortical projections do not. **C**. Flat map showing the projection strength of ipsilateral cortical inputs to the claustrum for intra-claustrum injections. Injections in the claustrum reveal descending projections from nearly all cortical areas with the notable exception of the primary visual area (Area V1) and the somatosensory area (Area 1) indicated in the figure. The exceptionally strong connections between the cerebral cortex and claustrum, as well as comparable strong long-distance intrinsic claustral connections, provide the infrastructure for the claustrum to mediate wide-ranging interactions across the cerebral cortex (for details see [84]). Panels B and C derived from data in [84].

There are three main channels through which cortical areas in primates can exchange signals: cortico–thalamo–cortical loops [83]; direct cortico–cortical pathways [2,20]; and cortico–claustral–cortical loops [84]. While projections across the cortex via the thalamus may be partly restricted to neighboring areas [85], cortico–cortical projections provide direct links between any two-thirds of cortical areas [20], and projections via the claustrum can directly influence virtually any cortical area [84]. To study these projections, tract tracing is used to define a weight index (FLNe) of connection strength in terms of the relative proportion of neurons participating in a particular cortical pathway. In addition to the high density of inter-areal communication via the claustrum, the weights of inter-areal interactions via the claustrum is significantly higher than those from inter-areal cortical connections. This is because direct cortico–cortical pathways exhibit an exponential decline in connection weight [86], which is not the case of projections via the claustrum hub [84] (Fig 2A and 2B).

Inspirationally, Crick and Koch [87] envisaged a special role for the claustrum in the inclusive nature of consciousness that allows you, “when holding a rose, to smell its fragrance and see its red petals while feeling its textured stem with your fingers.” These authors reasoned that binding information across modalities would require widespread claustral–cortical connectivity. While such connectivity was largely confirmed by numerous anatomical tracing investigations in rodents, these studies also claimed that cortico-claustral pathways were topographically organized [88]. Likewise, physiological experiments exploring sensory responses in claustrum described them as localized with largely unimodal responses and therefore seemingly at odds with the binding function, previously proposed by Crick and Koch [87,89–92]. In these investigatiions the apparent lack of multimodal sensory integration could be due to an absence of TD engagement [93–95]. Evidence that this might be the case comes from recent electrophysiological findings in mice suggesting functional convergence from widespread cortical sources onto single claustral neurons, establishing networks leading to multimodal sensory responses [96]. These findings suggest that a reappraisal of claustral function in higher cognitive processes including aspects of consciousness may be warranted [97].

Higher cortical association areas via claustral pathways to early sensory areas in macaques [84] can exert TD cognitive control [89]. In macaques, recent findings show that claustrum and insula share connectomic and transcriptomic features [84], and functional MRI (fMRI) studies suggest a possible involvement of insula in precision weighting of prediction errors in visual cortex [98]. We argue for a special role of the claustral-insula complex in long-distance interactions in the cortex, suggesting that it likely complements and prolongs direct inter-areal pathways in relaying TD predictions (Fig 2A–2C).

The claustrum receives input from ascending projections that control brain-state transitions in the wake–sleep cycle [99,100]. Activation of the claustrum leads to feedforward inhibition in the cortex via claustral projections onto inhibitory cortical cells [101], provoking a transient down state followed by a widespread synchronized down-to-up state transition across cortical areas, typical of activity patterns linked to slow-wave sleep [99]. Sharp wave ripples in the claustrum of rodents and its putative homolog in reptiles have a causal role in relaying and generating cortical state transitions found in slow-wave sleep [99,100]. The involvement of claustrum in state transitions in sleep, however, does not preclude an involvement in wake states, especially given that significantly higher levels of claustral neural activity are reported in wake versus sleep states [102,103].

Brain-state transitions are tightly correlated with specific behavioral repertoires [104–106], depend on the coordinated fluctuation of single-cell properties, and engage key brain functions across neocortex and hippocampus [107]. Rapid state transitions and their spatial distribution according to behavioral needs is a major and, as yet poorly understood, feature of the brain. A working hypothesis is that the spatial embedding of the claustrum and its intrinsic circuits [108] give it a privileged role in orchestrating rapid changes of brain state [109], promoting robust representation of sensory stimuli [110], and influencing TD signals [93]. This issue can be addressed in NHP using a combination of multi-site, high-yield, high-density electrophysiological recording of identified neurons in neocortex and claustrum during brain-state transitions in macaques engaged in cognitive tasks (see section on Roadmap for coordinated parallel investigations of NHP electrophysiology and human neuroimaging).

Recent findings in mouse show that the influence of the claustrum on the cortex is layer- and cell-type dependent [111]. These electrophysiological findings support the proposal that claustrum is involved in salience processing (i.e., directing attention to relevant, that is, salient, events) [112–114]. The interaction between salience and attentional mechanisms is mediated via precision weighting [115]. The cortex-wide connectivity of claustrum is complemented by a spatially extensive intra-claustral inhibition [108,116]. Hence, salience processing can be achieved by the cortico–claustral–cortical loops acting as a winner-take-all circuit [117], where the sensory stimulus of the winning claustral–cortical loop is the one that is selectively attended to [118,119] or becomes conscious [87]. This functional model is compatible with the evidence that the claustrum has a role in ensuring cognitive control [120,121].

Inter-regional long-range connections in the primate cortex fulfill a specific role in terms of the complexity of the structural network and their functional dynamics [122–124], and have been explored in large-scale dynamic models of cortical function [125,126]. Inter-regional connectivity in the cortex obeys an exponential distance rule (EDR), where connection weight declines approximately exponentially with distance [86]. The evolutionary expansion of the primate brain could contribute to making the critical task of the long-range pathways more fragile [80]. These structural limitations on long-range cortical interactions do not apply to cortico–claustral–cortical loops, as shown by our comparison of the intra-cortical and claustral-cortical connectivity in macaque (Fig 2B) [84]. Furthermore, this study of whole-brain connectivity and single-cell spatial transcriptomics (Box 1) of macaque claustrum revealed a regionalization with preferential cortical projections and differential composition of transcriptome-defined glutamatergic cell types, some of which may be macaque specific. There is also considerable evidence of the importance of the thalamus in precision weighting [127]. Importantly, the patterns of thalamic connectivity of the claustral sub-regions mirror those of the related cortical regions, allowing the ascending thalamic pathways to both structures to optimize precision weighting [84]. The rich thalamic connectivity of the macaque claustrum could imply that this structure has a specialized role in precision weighting.

The non-compliance of macaque claustral connectivity with the EDR may be highly relevant to recent findings of high-level prediction errors in early visual cortex. Multi-unit recordings showed that in macaque area V1, the primary visual area of the cortex receiving the bulk of the ascending visual information, natural stimuli with low predictability induced late-onset beta synchronization, reflecting cortical feedback [128]. Similar findings were found in humans, where fMRI results suggest that predictions are computed at higher cortical levels and consequently sent down the cortical hierarchy reaching area V1 [129,130]. Multi-site recordings in claustrum and cortex, paying particular attention to cortical area, layer, and cell type [111], and exploiting current understanding of macaque claustral cell types and regionalization [84], will be needed to clarify the role of claustrum in broadcasting predictions across the cortical hierarchy.

## A precision-weighting hub model of macaque claustral function

Our proposition that the claustrum serves as a precision-weighting hub builds on findings of Mathur and colleagues [120] suggesting that the claustrum is a task-dependent hub whereby higher-order areas dynamically instantiate task-relevant cortical networks in support of cognitive control. This view extends earlier conceptions of the claustrum as a conductor of cortical binding, an integrator of synchrony, and a mediator of attentional selection [87,118,131]. These formulations suggest a predictive processing framework, in which attentional selection is often understood as the context-sensitive precision weighting of prediction error signals, and cognitive control may depend on the allocation of precision over task-relevant representations and policies. Our proposal is further motivated by recent single-unit recordings in humans during aversive learning, showing that claustrum neurons track model-derived uncertainty and prediction error, two variables well suited to support precision weighting [132]. Here, we describe two aspects of the anatomical organization of the macaque claustrum that suggest differential modulation of the gain of prediction-error signals via precision weighting across the entire cortical hierarchy.

### Claustrum extends the reach of TD inter-areal contextual signaling

**Fig 3:**
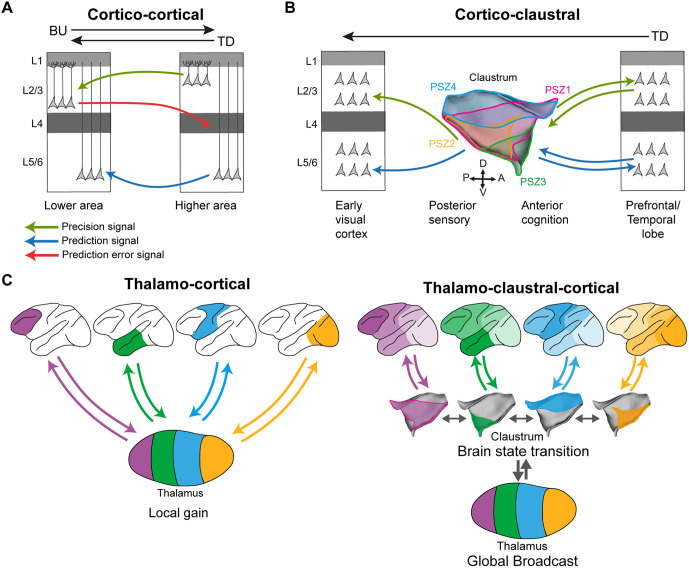
A model of claustral precision-weighting function. **A**. Inter-areal connectivity in terms of hierarchical predictive coding (hPC) theory. **B**. Empirically observed claustral–cortical connectivity in terms of hPC theory. **C**. Organization of thalamo–cortical loops (left) and thalamo–claustral–cortical loops (right) suggests complementary broadcast functions. Panels B and C adapted from [84], laminar connectivity of panels A and B derived from data in [84,133]. BU, bottom up; L1-6, layers 1-6; PSZ1-4, claustral subdivisions 1-4; TD, top down.

The claustrum can be thought of as a specialized network exerting a TD influence on the inter-areal cortical network [87,118,120,131]. In the inter-areal network, TD projections from supragranular layers are thought to carry precision signals, while those from infragranular layers carry TD predictions; this contrasts with the BU projections in the supragranular layers that target layer 4 (L4) and carry prediction errors [37] (Fig 3A). The anatomy of the claustrum shows that, with respect to the inter-areal cortical network, it has a strong TD bias: posterior sensory cortex and, in particular, early visual areas, have weak or no input to claustrum, contrasting with frontal regions, including the temporal lobe, which have strong input from both upper and lower layers [84] (Fig 3B). These differences in the projections from posterior and rostral cortex to claustrum contrast with the projections of claustrum to cortex, being moderately strong to all hierarchical levels (Fig 2).

The TD claustrum-mediated precision-weighted model supported by cortico–claustral interactions (Fig 3A right) leads to falsifiable predictions that distinguish it from classical hPC accounts. First, perturbing claustral activity in mismatch or oddball paradigms (Box 1) should selectively alter the gain of prediction error responses in sensory cortex [134]. Second, the model supports a directed functional pathway from higher-order cortices to sensory cortex [135], so that precision and prediction signals in frontal areas should precede and modulate sensory cortical responses. This temporal sequence, where frontal regions lead claustral activity, which in turns precedes sensory cortex modulation, should be detectable using high-density laminar electrophysiological recordings and causal analysis. Third, recordings along the anterior–posterior axis of the claustrum should reveal sensory precision in posterior claustrum and cognitive precision in anterior claustrum. In this manner, posterior claustrum should correlate with sensory uncertainty and anterior claustrum with cognitive control [120]. Finally, multi-site recordings in claustrum and frontal and sensory cortex should reveal claustral coordination of large-scale cortical synchrony, particularly during difficult tasks requiring precision modulation. Here, claustral activity is expected to correlate with cross-frequency coupling, and disruption of claustral function should impair long-range synchronization.

### Thalamic regulation of claustral broadcast

The thalamus has been implicated in higher cognitive function both from a connectionist and from a predictive coding perspective [136]. There is considerable evidence that contextual information in higher order thalamic nuclei, but also in thalamic relay nuclei, activate the cortex via thalamo–cortical pathways [85,127,137–142]. The contextual information in the pulvinar nuclei (Box 1) and its mouse homologue has both a *trans-*thalamic origin, as well as being derived from descending projections from the higher order areas of the cortex [140,141,143]. Recent work has identified activation of area V1 via a pulvinar-driven canonical VIP → SOM disinhibitory motif [142] (Box 1), showing that thalamo–cortical pathways might exploit this conserved interneuron architecture to modulate cortical gain. Given the ubiquity of VIP–SOM circuitry across the cortical hierarchy [144,145] and its established role in cortico–cortical TD control [144–147], the pulvinar–VIP–SOM could constitute a circuit principle through which the thalamus gates cortical processing in a behaviorally relevant context-dependent manner. The claustrum, like the cortex, appears to possess both the circuitry and the connectivity needed to support thalamic-gated contextual modulation. Claustral VIP interneurons can disinhibit projection neurons via local inhibitory motifs [148]. In addition, large-scale tracing studies indicate that the claustrum receives a rich input from thalamic nuclei [84,149]. Together, these findings support the hypothesis that ascending thalamic signals could provide contextual information to the claustrum by engaging a local VIP–SOM-like disinhibitory circuit, although the specific causal mechanism remains to be determined experimentally.

We propose that thalamic inputs to the claustrum provide domain-specific control signals that regulate the gain and temporal regime of claustral activity, rather than conveying detailed representational content. Consistent with the finding that claustral subdivisions are preferentially connected with distinct cortical systems and receive matched projections from corresponding thalamic nuclei [84], we suggest that this thalamo–claustral system operates synergistically with thalamo–cortical pathways and that the two pathways serve distinct computational roles (Fig 3C). Whereas thalamo–cortical loops might modulate gain locally within individual cortical circuits, thalamo–claustral interactions are organized as parallel, sector-specific loops, so that thalamic input may determine the broadcast capacity of the corresponding claustral subnetwork, and cortico–claustral projections stabilize this regime via closed-loop dynamics. We therefore hypothesize that claustrum functions as a mesoscale conduit allowing thalamic input to regulate the precision or effective weight of claustrum-mediated cortical interactions, enabling flexible, context-dependent orchestration of distributed cortical processing.

An important mechanistic component of this system is provided by the local inhibitory circuitry within the claustrum. Recent work by Augustine and colleagues [148] demonstrates that claustral inhibitory circuits act as spatial nonlinear filters of incoming excitation. This leads to the experimentally verified prediction that claustral contributions to behavior are most prominent under noisy conditions, during distractor processing, and in early stages of learning when task-relevant neural ensembles are not yet established [119,120,150]. We suggest that such spatial noise filtering can be interpreted as a circuit-level implementation of precision weighting. Rather than passively suppressing background activity, claustral inhibitory circuits might resolve competition between relevant and irrelevant representations, enabling selective amplification of task-relevant signals while suppressing competing inputs.

This model of the role of thalamus on claustral function generates testable predictions. First, perturbation of the thalamic input to a particular claustral sector should selectively alter the influence of that sector on its corresponding cortical targets, without uniformly affecting other claustral outputs. Second, such perturbations should primarily modulate the gain, synchrony, or reliability of claustrum-driven cortical activity, rather than its representational content. Third, functional coupling between claustrum and matched thalamic nuclei should increase during behavioral states requiring rapid reprioritization, such as attentional shifts, conflict, or unexpected sensory events. Finally, manipulation of thalamo–cortical and thalamo–claustral pathways stemming from a given thalamic nucleus should reveal complementary effects, with notably local versus distributed modulation of cortical communication.

An important comparative issue concerns whether claustrum forms a reciprocal loop with thalamus across species. Several rodent studies suggest that projections from claustrum to thalamus are weak or absent [149,151], whereas findings in macaques are more consistent with robust claustral–thalamic connectivity [84,152]. These observations could imply that, in rodents, thalamic influence on claustrum is likely to be implemented mainly in a unidirectional manner, biasing claustral gain, state, or broadcast in the absence of a recurrent thalamo–claustral control loop. By contrast, reciprocal claustro–thalamic connectivity in primates would permit a richer architecture in which claustrum is not merely regulated by thalamus but participates with it in iterative coordination of distributed cortical processing. From this view, a basic thalamus-to-claustrum mechanism may be evolutionarily conserved, whereas a recurrent thalamo–claustral contribution to precision weighting could be enhanced or possibly specific to primates.

## Convergence of BU and TD signals at laminar scale in the cortical hierarchy

**Fig 4:**
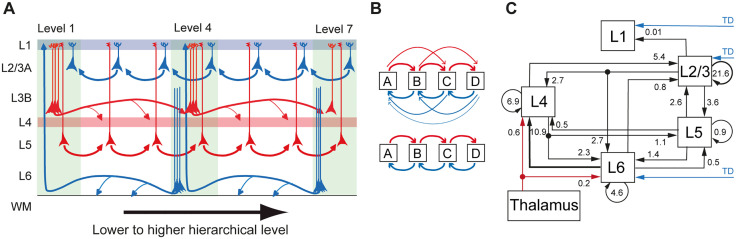
Direct cortico–cortical pathways. **A**. Dual counter-stream inter-areal architecture, is a conspicuous anatomical feature of primate cortex in which two streams of bottom-up neurons (red) and top-down neurons (blue) connect high to low areas**.** However, because fibers of passage obscure the description of these circuits, crucial details of the connectivity are missing: retrograde tracer injection have revealed the laminar distribution of cells of origin to an area, but not to an individual layer; anterograde tracer injections have revealed the laminar distribution of projection terminals from an area but not from a particular layer. Globally, layer 6 (L6) TD signals are thought to target mostly infragranular layers over short distances, and L1, L2, and L3 over longer distances. For a detailed description of what needs to be further investigated in the dual counter-stream architecture see [93]. A major advance would be to overcome these shortcomings using monosynaptic viral tracing and single-unit recording. **B**. Schema of inter-areal connectivity, top panel summarizes empirically observed connectivity where each area projects to multiple levels/areas but more strongly to adjacent levels/areas. Bottom panel shows the serial connectivity incorrectly assumed to exist by numerous theoretical accounts of cortical processing. **C.** Canonical local circuit of BU and TD connections between hierarchical levels. Panel A adapted from [20], panel B adapted from [93], Panel C adapted from [154], Copyright 2004 Society for Neuroscience 0270-6474/04/248441-13.00/0. WM, white matter.

In a recent review of inter-areal processing, we argued [93] that the understanding and modelling of the interaction of BU and TD streams need to be better constrained and informed by a greatly improved understanding of the hierarchical circuits that are involved in their integration (Fig 4). There are fundamental differences between BU and TD connectomics: they constitute strictly separate populations, supportive of organized inter-areal interactions likely to subserve the integration of priors and sensory evidence; BU and TD form multiple pathways, originating from different laminar compartments (Fig 4A); and single brain areas project to multiple hierarchical levels (Fig 4B upper panel), requiring a revision of the serial processing scheme of traditional hPC models [37,153] (Fig 4B lower panel). This latter point suggests that, in order for interaction of BU and TD signals to occur, it is necessary for activity to be expressed, assembled, and integrated over a large, distributed recurrent network.

Hence, future anatomical tracing in NHP will need to investigate, for the first time, the weighted layer-to-layer connections between cortical areas. This will provide critical data for developing computational models of the circuit on how multiple hierarchical levels interact with each other and integrate BU and TD information, supporting cell-type layer-specific TD and BU processes.

One major difficulty with the active inference model is the glaring lack of detail concerning hierarchical circuits (Fig 4A) that ensure direct interaction between BU and TD pathways. Hence, the current understanding of anatomical cortical circuits lags behind the sophistication of the theoretical framework of active inference [93]. Existing accounts of the circuits of hierarchy as described in Fig 4 are relatively crude, being entirely based on laminar patterns of connectivity and mesoscale activity measures [32,93,155].

Moreover, the generation of prediction error and its modulation by precision requires an interaction between BU and TD pathways. Presently, we lack empirical evidence concerning the circuits implementing such interactions. TD signals from supragranular layers may largely but not exclusively target upper layers and, in particular, layer 1 (L1) [156]. The laminar targets of the more extensive L6 TD signals are even less certain, but at least over long distances they appear to project to L2/3 and L1 [20,156–160]. One hypothesis [45,161] is that L6 neurons project TD to L6 neurons at lower levels. Such L6 > L6 TD signals have been reported [162]. Within the same cortical column, L6 neurons project to L4 and L2/3 [154]. Shipp [45,163] speculates that such local L6 > L2/3 connections would enable the L6 TD pathway to participate in generating prediction error in L2/3. However, another possibility that needs to be considered is that the TD signal into upstream L6 is relayed locally to L4, a connection which is substantially stronger than the local L6 > L3 signal [154] (Fig 4C). Hence, there are three routes by which TD excitatory influence can interact with BU signals located in the granular and supragranular layers, one direct and two indirect routes; these three routes may employ distinct cell types and could subserve distinct functions. Alternatively, TD pathways may interact with BU signals via the local inhibitory microcircuit of the upstream triggering disinhibitory and inhibitory processes underlying respectively prediction error and precision-weighting of prediction error [75,164]. Note that the TD route via L6 and targeting L4 corresponds to that proposed by Rao and Ballard in their original description of hPC [36].

L1 is a major target of TD pathways [62], and there is now strong evidence that the TD projection to L1 can serve to control BU–TD interactions via contacts upon the apical tufts in L1 belonging to the pyramidal neurons located in L5 and L2/3 [65]. In line with these findings is the recent characterization in mouse of an excitatory cell type called Baz1a in L2/3 [165], with a demonstrable capability to integrate TD and BU activity. Baz1a generates prediction errors in area V1 in mice [166]. L2/3 in primates is known to house five distinct glutamatergic cell types [167–170], two more than in mouse, suggesting an enriched interaction of TD and BU influence in NHP and human cortex. Interestingly, two of the cell types described in human L2/3 are homologous to cells in mouse L5/6 [167]. Could such cells constitute the targets for the within-column L6 neuron projections? To summarize, we lack a clear understanding in primate of how the circuits of the more substantial L6 TD signals differ from those of the L3 TD signals, nor is it clear if L6 signals to L4 and L3 have distinct roles in terms of integration of TD signals. Such issues will be an important focus for future research.

The literature on cortico–claustral–cortical loops reveals only a few studies using anterograde tracers to examine the projections of claustrum to the cortex in NHP. However, given the global similarity of cortical connectivity in macaque to that observed in cat, we can consider the numerous studies in cat on claustral projections to the cortex. Three studies using autoradiography have shown claustral projections to all layers of cat visual cortex with notably higher densities in L4 and L6 [91,171,172]. These findings suggest that the claustral input to sensory cortex is a major driving input, although a study using biotinylated dextrin amine found claustral projections concentrated in supragranular layers [133]. Overall, these findings are intriguing because they suggest that, at least in cat, the cortico–claustral–cortical loop could provide a direct interaction between BU and TD signals, which is not the case for direct cortico–cortical pathways.

## Evidence of TD prediction and BU prediction-error signals

Testing different models of perceptual inference requires physiological investigations in the awake brain combined with precise psychophysical control. Here, the major challenge is to distinguish experimentally between BU and TD processes in vivo. An additional challenge is to determine the computational content of putative mismatch signals, since similar response profiles can arise from distinct mechanisms [1]. While BU information is relatively easily manipulated via external stimuli, TD information is generated internally and can only be indirectly manipulated.

### TD prediction signals

Physiological investigations have shown profound effects of TD information on neural activity in early visual areas [59,173–176]. In particular, investigations in humans suggest that, during imagery, imagined shapes can be decoded and visually reconstructed from fMRI activity in area V1 [60,177,178]. Furthermore, experiments where part of a natural scene is occluded show that neurons in early visual areas with receptive fields inside the occluded part are activated in specific laminar patterns, decoding contextual information with respect to the occluded image [179,180]. Recent work in NHP and mice also suggests that TD projections cause working-memory-specific activations in early visual cortex [59,181]. Interestingly, stimulus predictability enhances synchronization of neurons, suggesting distinct signaling mechanisms for surprising versus correctly predicted information to higher brain areas [182]. Finally, recent analysis of high-density laminar recordings in mice showed that prediction-error signals are more tuned to higher-level features and long-term priors than to low-level features, even in area V1, suggesting a TD origin [128,130,183]. Altogether, these findings point to the need for more detailed physiological investigations during perceptual tasks.

There is only partial empirical evidence for TD predictive signals, but also for how predictions are represented, where mismatches are computed, and whether putative error signals update internal models in the manner assumed by classical predictive coding [1]. Rao and Ballard [36] hypothesized that TD projecting neurons in L5/6 would maintain an internal representation for generating predictions of lower-level activity. However, the empirical evidence for the laminar location of prediction does not completely fit with the Rao and Ballard model. In mice, a small contingent of prediction units have been recorded, but in L2/3, not L5/6, although these units are driven by TD inputs [184]. In both NHP and humans, memory recall and prediction signals have been recorded in L5/6 [185,186], and empirical evidence supports that they are conveyed by TD pathways [51,187,188]. Rao and Ballard proposed that a second group of neurons in L2/3 calculate prediction errors to be transmitted to the higher levels. There is empirical evidence of different types of neurons encoding prediction error in L2/3 in mice [166,189] with only minute proportions in L5/6 [190], and prediction-error signals have been detected in L2/3 in primates [191]. More recently a third group of neurons was proposed to relay a TD precision signal associated with attention [46], which is hypothesized to estimate the reliability of the sensory evidence and to weight the ascending prediction-error signal accordingly [37,192]. Theoretical grounds suggest that precision is implemented by cholinergic modulation of prediction-error signals [193], and recent empirical evidence for this has been obtained in infragranular layers of rat auditory cortex [194] and with fMRI in humans [134]. Contrary to prediction error and prediction signals, and despite the theoretical importance of such a signal, we know of only one report claiming a potential TD precision motif [162], although there are strong candidates for such a signal [195,196].

The theoretical framework described above corresponds to classical hPC as formulated by [36], with a cortical implementation as described by [37]. There are a number of putative algorithms serving hPC [47,197]. A body of experimental evidence points to the importance of L1 as a target of TD influences. Because apical dendritic tufts of supragranular pyramidal neurons are located in L1, it has been proposed that they process TD signals while the basal dendrites of these cells process BU signals [65,198]. Recently, an organizational framework based on the supragranular pyramidal cells led to the development of a theory of hierarchical dendritic predictive coding (hdPC) [199–201], which differs substantially from hPC in so far as errors and predictions are supposedly handled by the same cell, and which will need to be addressed in future investigations. We anticipate that anatomical and functional studies will yield important data to test predictions of both hPC and hdPC theories.

Is there a particular difficulty in finding evidence of hPC in the primate brain? The computational architecture for the hPC of Rao and Ballard [36] requires TD generation of spiking activity [202]. TD prediction-error spiking in macaques has been reported in several visual spatial paradigms. Repeated local and global stimuli give rise to strong prediction-error spiking [51,203]. However, it appears that in NHP, TD generation of prediction-error spiking in early sensory cortex is most reliably observed in response to local oddball stimuli [51,188,202–204]. It has been suggested that local oddball response may confound prediction error with an adaptation response [202]; however, in hPC, adaptation is not necessarily a separate process but could reflect a local reduction in precision [193,205], so it does not follow that local oddball spiking alone in early sensory cortex can be conceived as the reflection of ‘low-level’ TD predictions [206]. In any case, adaptation can conceivably be accommodated in claustral precision function via a capacity to contextualize local adjustments of precision in early cortical areas [207].

In mice, recent evidence demonstrates that the local oddball response corresponds to TD prediction-error signals and that global oddball response in sensory areas involves a specific cell type [208]. In future experiments, appropriate controls will need to be used to disambiguate local oddball from adaptation [82]. In macaques, in contrast to the TD local oddball response, robust spiking activity to global oddball stimuli is largely restricted to high-order cortical areas [51,203], and here, restricted spiking could serve to limit cognitive penetration in early sensory cortices [209]. With regards to the view that increasing the precision of selected prediction errors empowers them to revise higher-level representations, one could conceive this as a kind of ignition, as seen in global neuronal workspace theories of consciousness [210]. Altogether, these findings confirm the importance of TD prediction signals and suggest that spiking activity following local oddball stimuli found in macaque sensory cortical areas differs significantly from the higher-order areas’ spiking activity, following global oddball stimuli.

### The physiology of BU and TD integration

Communication between cortical areas is thought to be influenced by the temporal structure of signals, which can be selectively processed based on resonance or entrainment mechanisms [211,212]. Based on investigations of cortical dynamics across the hierarchy, three kinds of regularities in the relation of function to structure have been identified. First, during active states, time scales of aperiodic and rhythmic activity tend to be progressively slower in higher hierarchical levels [126,212–214], presumably reflecting the increased integration of information across time that is necessary for the maintenance of sensory predictions and the higher cognitive functions they support. Second, cortical areas can express different dominant frequencies in a given behavioral condition (e.g., beta in parietal cortex and gamma in occipital cortex [215,216]), referred to as ‘frequency-specific networks’ [201,215]. Indeed, Hoffman and colleagues [216] showed the identity of the brain area can be uniquely decoded from its local field potential spectrum, illustrating the intrinsic heterogeneity of dynamics across cortical areas. This heterogeneity might in part reflect hierarchical gradients [217–219], but the dependence of temporal dynamics on stimulus drive and other factors can further increase the divergence in frequencies. Furthermore, a gradient in time scales and oscillation frequencies could be an emergent phenomenon in a hierarchical system performing predictive processing [220]. Third, several studies have shown that low-frequency rhythms tend to be more prominent in deeper layers of the cortex, while superficial layers tend to express higher frequency rhythms [221–223], although it is debated how systematic and strong this laminar distinction is [201,224–226]. Cell types encoding predictions reflect a linear accumulation of prediction errors and, conversely, prediction errors are non-linear functions of predictions [37], hence it has been proposed that the characteristic frequencies of TD and BU signaling constitute emergent properties of hPC non-linear generative models [220].

These structure–function relationships, in particular the distribution of frequencies across the hierarchy and layers, naturally imply that a given cortical area might receive inputs at lower frequencies from higher hierarchical levels and higher frequencies from lower hierarchical levels, which might differ from the area intrinsic frequency [32,155,212,227]. A frequency separation could confer functional advantages, in so far as TD and BU streams of information are separated in the frequency domain. A cortical area might thus ‘interpret’ the hierarchical origin of an incoming signal based on its spectral signature, enabling the area to process the input either as a prediction or as an error signal. Frequency separations of this sort raise important questions as to how TD and BU information streams are effectively integrated, especially considering the fact that hierarchy is not serially organized and a given area will receive inputs from tens of other areas with a wide range of frequencies.

Various theoretical frameworks such as communication-through-coherence or resonance assume that communication between areas takes place within a shared frequency band. However, the integration of a highly divergent set of frequencies cannot be accounted for by such theoretical frameworks [212]. Non-linear integration mechanisms are therefore essential to understand the integration of BU and TD signals [212]. These non-linear mechanisms could help establish stable relationships between areas, leading to an integration of information across frequencies. Such non-linear integration mechanisms might reflect the rich repertoire of non-linear integration within single neurons, dependent on voltage-gated channels like NMDA receptor [228,229], combined with emergent non-linear dynamics produced by the recurrent dynamics in the local circuit. In this light, it is important to note that initial theoretical models of hPC rest essentially on largely linear dynamics, raising the question of how local circuits integrate TD and BU signals through non-linear, recurrent dynamics. This holds true both for classic hPC [36] and hdPC [199], as discussed in the previous section.

However, there is a strongly non-linear integration of TD inputs into the apical dendrites with back-propagating action potentials and baso-dendritic inputs. The resulting calcium and NMDA spikes generated in the apical dendrite can lead to a massive non-linear amplification of apical inputs and changes in firing mode from single to burst spikes [228,229]. This calls for theoretical models of hPC that incorporate non-linear integration mechanisms [230]. The computation of the difference between sensory predictions and evidence may depend on attractor-like dynamics in recurrently coupled systems that control the interaction of BU and TD inputs [231]. This attractor can be defined by variational free energy minima [28], where the precision-weighted prediction errors supply the variational free energy gradients, and BU and TD input interactions are mediated specifically by changes in precision. Several studies highlight that non-linear long-range interactions can show stronger modulations with behavioral and perceptual states compared to linear interactions such as coherence [212,232–234]. Furthermore, prediction-error signals across the cortical hierarchy are not redundant but rather convey the synergistic information that results from recurrent interactions between areas [235]. These considerations point to a critical need to examine the non-linear interaction and integrations between signals from different areas and laminar compartments during perceptual tasks.

## The need for a cell-type account of BU and TD signals and circuits

Many uncertainties concerning the circuitry underlying prediction, prediction error, and precision stem from the current inability of classical investigations to disentangle the BU and TD circuits in the dual counter-stream architecture and their integration into the local circuit [93]. However new tools are at hand. One that should enable major progress in deciphering neuronal circuits is transcriptome-based cell-type classification, which will lead to better understanding of the particular roles of different circuit components. With respect to the canonical circuits of hPC, high-throughput transcriptome-based methods for cell-type identification in rodents [236] have been shown to scale to primates [31,168,170,237]. Defining cell types by their transcriptomic profiles enables the identification of cell-type specific short gene-regulatory enhancer sequences that can be used to develop adeno-associated virus (AAV)-based vectors for cell-type selective genetic manipulation, thereby extending to primates certain viral tools that have proved uniquely productive in the mouse [238]. Recent work using these methods has identified a wide variety of ‘new’ excitatory projection neuron types, enabling finer classification of the population of cortico–cortical, or intratelencephalic (IT) neurons, subsets of which give rise to BU and TD projections [168,237,239–241]. This work has revealed an evolutionary expansion of the diversity of IT types in L3 of primate cortex [167–170] that warrants further intensive investigation.

A cell-type account of hierarchical anatomy and physiology in NHP is now made possible by the recent description of the cell-type organization of the macaque cortex via spatial transcriptomics [31]. A critical element will be to perform genomic analysis of the BU and TD projection cell types and the circuits that they form in extrastriate visual cortex of NHP to uncover their functional diversity. This will lead, in turn, to the development of enhancer-AAV tools for not only mapping but experimentally manipulating the projection cell types responsible for canonical pathways of hPC. In this manner, future electrophysiology recordings will be able to identify the functional properties of TD and BU pathways. Here, this approach is greatly facilitated by the recent description of cell-type-specific and primate-selective enhancers in macaques [242].

## Roadmap for coordinated parallel investigations of NHP electrophysiology and human neuroimaging

An overarching objective of neuroscience is to understand hierarchical processing in the human brain, which in the emerging field of computational psychiatry is expected to be key to understanding numerous brain disorders [243]. hPC theories hold strong explanatory power and have been proposed to constitute a potential ‘paradigm shift’ [244], as witnessed by their successful application in mouse models. However, accumulating evidence demonstrates numerous primate-specific features during development that determine the unique cognitive architecture of the primate cortex [79,245]. These primate-specific features explain why mice are often inadequate models for human neuropsychiatric diseases [78]. Even structural properties alone constitute a barrier for extrapolation: the mouse cortex exhibits a graph density of 97%, far exceeding that of NHP and human cortex [80,246], which implies the presence of numerous inter-areal connections in mice that have no primate homologs. One example is the projection from the anterior cingulate cortex to area V1 in mouse, which conveys TD spatial and stimulus-specific predictions [184], yet is absent in NHP. Similarly, the aforementioned primate-specific expansion of glutamatergic cell types in L2/3 [167,168] reflect unique developmental processes [79] that further differentiate primate cortical computations from those of rodents.

Having established NHP as the appropriate model for probing primate cortical mechanisms, the literature reviewed in the previous section nevertheless remains fragmented and isolated, lacking a common framework for a mechanistic understanding of TD and BU processes across scales. To overcome this limitation, we propose a coordinated parallel investigation is needed that integrates NHP electrophysiology and human neuroimaging, capitalizing on the strengths of each modality while compensating for their intrinsic limitations. Motivated by the goal of understanding primate cortical computation, this combined approach could be used to test hypotheses about TD and BU processes within the hPC framework, targeting cortico–cortical and cortico–claustral laminar pathways both anatomically (e.g., cell types) and functionally (e.g., relevance for perceptual inference, frequency-specific contributions).

**Fig 5:**
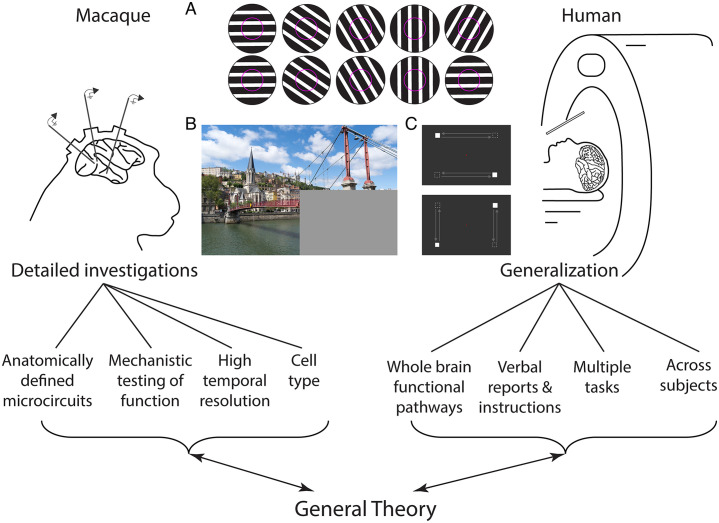
Advantages in comparing functional signals obtained from humans and non-human primates during similar visuo-cognitive tasks. **A.** Sequences of grating stimuli with orientation temporally predictable from repeated sequences in a local–global oddball paradigm where the fifth stimulus naturally follows the preceding sequence (top) or skips one orientation (bottom). The local oddball is the fifth stimulus of the bottom sequence (presentation in block, for which the bottom sequence constitutes 20% of trials and the top sequence 80%). The global oddball is obtained by reversing the proportion of the two sequences (top sequence presented in 20% of trials and bottom sequence in 80%), where the fifth stimulus on the top sequence becomes the global oddball. **B**. A restricted region of a visual scene is patched in the occlusion paradigm such that feedforward signals of the occluded part do not reach the corresponding retinotopic representation of the early visual areas studied. Image from Jean-Christophe BENOIST, CC BY 4.0 https://creativecommons.org/licenses/by/4.0/, edited with gray area for occlusion. **C.** Perceptual switching between apparent horizontal (top) or vertical (bottom) motion is created by alternating frames containing two non-moving squares. Panel C adapted from [249].

To experimentally manipulate TD–BU interactions, it will be necessary deploy not one but a battery of visual paradigms designed to reduce retinal input and induce internally generated activations under different TD conditions. A review of paradigms previously used in either NHP or humans reveals numerous paradigms covering a broad range of TD effects targeting early visual cortex, notably including area V1. Examples include: motion-specific illusions such as apparent [247,248] and bistable motion [176,249], which are hypothesized to involve TD influences originating from motion-sensitive areas, such as the middle temporal area; illusory contours such as the Kanizsa illusion [175], carried by TD input from the secondary visual area, area V2 (in NHP) or lateral-occipital area (in humans); long-range color filling-in [250–252] carried by TD projections from ventral and dorsal pathways; and finally occlusion paradigms [179,180] that convey contextual TD signals from V2/V3 (second and third visual areas of the cortex) and higher areas. All TD conditions will therefore need to be balanced in luminance and background color, paired with corresponding control conditions, and presented in identical retinotopic locations. For example, both the apparent motion and Kanizsa contours could be presented on a uniform gray background and be displayed in identical locations of the visual field. This would ensure that differences between conditions can be directly attributed to TD manipulation rather than inadvertent engagement of distinct neuronal populations. Such a coordinated design would impose cross-species constraints (Fig 5). The retinotopic location of NHP electrode implantations could be used to define the visual field, where all stimuli are presented in both species. To maximize feasibility across a large task battery for NHP training, all paradigms would need to rely on passive fixation rather than explicit behavioral reports, and adaptation effects minimized using simple detection tasks (saccades in NHP; button presses in humans).

For the proposed investigation, the human cohort should follow a deep-sampling strategy [253], acquiring extensive data from a small number of individuals (5–10), mirroring the NHP approach with two or three animals. To resolve issues concerning laminar-specific cortico–cortical and cortico–claustral connectivity, ultra-high-field (≥7T) fMRI would be an optimal technique, offering sub-millimeter resolution capable of resolving mesoscopic structures such as layers and columns [254–261]. Despite multiple layer-fMRI studies probing TD effects in area V1 (e.g., [175,262]), findings vary across studies, partly due to differences in acquisition modality (e.g., gradient echo, spin echo, vascular space occupancy; VASO) and contrast mechanism (blood-oxygen level dependent; BOLD, versus cerebral blood volume; CBV,-sensitive fMRI). VASO-based acquisition should therefore be favored for CBV-sensitive fMRI due to its higher specificity with respect to the microvasculature and its relative robustness against oxygenation-related vascular changes and large draining vein effects that can mislocalize neuronal activation [263–266].

When targeting the claustrum non-invasively with fMRI in humans, ultra-high spatial resolution provides a key advantage by improving the separation of signals from the claustrum and the adjacent insular cortex. Owing to its thin geometry and close proximity to surrounding structures (e.g., the insula), studies using conventional resolution (3–1.5 mm isotropic) are highly susceptible to partial volume effects and have therefore often relied on modelling or deconvolution approaches to disentangle claustral signals from those of neighboring regions [267]. By contrast, we advocate adopting sub-millimeter acquisitions (0.8 mm isotropic) at ≥ 7T to directly mitigate these limitations by reducing partial volume, thereby enabling a more faithful isolation of claustral signals. Similar acquisition techniques at 7T have been recently used for imaging visual features of the claustrum [268].

Moreover, a complementary source of signal mixing arises from the vasculature, particularly venous contributions that can dominate the fMRI signal. For instance, shared draining veins between the claustrum and the insular cortex can blur the measured signal, leading to spatial mislocalization of the underlying neuronal activity [249]. To address this, we propose leveraging recent advances in high-resolution vascular imaging, achieving up to 0.35 mm isotropic resolution, to explicitly resolve the vascular architecture of the claustrum and its neighboring regions [269]. This approach will enable a more informed interpretation of functional signals by improving the characterization of neurovascular coupling and enhancing the spatial specificity of inferred neuronal sources in this underexplored structure.

Inter-subject variability constitutes an additional source of inconsistency. A deep-sampling design could be used to address both challenges by holding acquisition parameters and subjects constant across all tasks; thus, observed laminar differences can more confidently be attributed to differences in TD modulation. A similar strategy was used by Bergmann and colleagues [262], who employed two paradigms (visual imagery and contour illusions) and revealed distinct area V1 laminar profiles: deep layer involvement for imagery and superficial layer involvement for contour perception. This approach could be extended by including more paradigms (3–4) while maintaining feasibility within 5–6 scanning sessions per individual (~10–12 hours).

Perception arises from distributed cortico–cortical and cortico–subcortical interactions, particularly when TD signals dominate under reduced BU input. This complexity motivates our suggestion for a parallel NHP and human approach. Human layer-fMRI, with its advantageous extensive brain coverage, can identify the main laminar correlates of perception across this extended network. The findings from such studies would then subsequently guide targeted NHP electrophysiology, which provides the temporal precision and anatomical specificity needed to dissect the underlying circuitry.

Finally, NHP recordings would need to ultimately build on the viral tracing techniques previously mentioned in order to express light-sensitive opsins in specific populations of BU and TD neurons across multiple levels of the visual hierarchy. This will enable opto-tagging of projection neurons by the precise identification of single and multi-unit light-induced electrophysiological responses from high-density laminar recordings. The exploitation of enhancer–AAV tools developed for tracing the microcircuits of prediction could be used to enable recording from identified BU- and TD-projecting neurons, as well as helping to identify BU and TD inputs to neurons. Based on the circuits described in NHPs, it might then be possible to build generative (e.g., dynamic, causal) models of the laminar pattern of neural activity, generating the fMRI signals to test the models in humans [270].

Our argument is that clarification of where the signals and circuits of TD and BU converge in the human visual cortex requires a concerted mesoscale approach that exploits molecularly defined cell types to trace circuits and dynamics in NHP aligned with ultra-high field fMRI in humans. In turn, resolving the where and how will, we believe, lead to a more profound understanding of why BU and TD convergence is such a crucial aspect of human brain function, potentially providing the key to understanding the relationship between mind and brain. However, we need to guard against the notion that classical hPC will necessarily provide an inclusive and conclusive account of the mind–world relationship. The known neurobiology of the cortex leads us to anticipate multiple, possibly circuit-specific, levels of prediction signals: weak (imprecise) prediction signals could correspond to a model-based knowledge-sparse implementation of hPC, in contrast to strong (precise) prediction signals, as suggested in a discussion between Anderson, Chemero, and Clark [43]. Furthermore, a range of TD prediction signals could also intermesh with other proposed models, including mixed generative-feedforward models [40], predictive routing [191], and hdPC [199], leading to a varying hPC phenomena across sensory systems [271] and species [272]. Finally, significant progress in primate hPC in terms of circuits and signals is likely to further emphasize the synergy between neurobiology and artificial intelligence, with important consequences for both domains [74].

## Conclusions

In this Essay, we investigated theories and models of perception and explored the empirical need for detailed descriptions of the circuits and signals supporting prediction, mismatch sensitivity, and precision-like control. We anticipate that this highly fertile area of empirical research may substantially refine current predictive processing theories. For example, long-term priors in area V1 are more strongly tuned to high-level features originating from upper reaches of the cortical hierarchy [128–130], suggesting that feature-specific predictive processing is a general feature of the cortex [183]. Interestingly, similar TD long-distance predictions identified in rodents have been shown to trigger prediction errors in primary areas [273,274]. These observations underline the values of cross-species comparisons, which can provide a deeper insight into the nature of the prediction signal and are expected to lead to a reappraisal of perceptual theories and models.

The precision-weighting hub model of claustrum that we propose shows that defined laminar projections of the cortex to claustrum could convey TD prediction and precision signals. The proposal that claustrum has a central role in precision unifies numerous properties of the claustrum, including orchestration of brain states and cognitive control. While predictive processing frameworks have been highly successful in formalizing brain function in terms of prediction error and precision, the biological implementation of precision remains unresolved. In contrast to the extensive empirical evidence for prediction-error signals, direct evidence for precision as a distinct neural quantity, particularly within TD cortical pathways, is comparatively sparse. We therefore propose a theoretical framework in which precision-like control might not be a characteristic property of the inter-areal network, but instead involve specialized circuit mechanisms involving thalamo–cortical and thalamo–claustral–cortical networks (Fig 3C). In this framework, thalamic inputs to cortex serve to calibrate the gain- and state-dependence of domain-specific processing streams, while the thalamo–claustral inputs to cortex implement spatially structured, inhibitory filtering that resolves competition between cortical representations and orchestrates their broadcast across multiple hierarchical levels. This division of labor provides a biologically grounded account of precision that extends beyond TD pathways alone and generates testable predictions linking thalamo–claustral–cortical dynamics to the flexible coordination of large-scale cortical activity under conditions of uncertainty, distraction, and learning.

An important implication of the framework proposed here is that the circuits supporting predictive processing and precision weighting might differ substantially across species. In particular, emerging evidence suggests that reciprocal interactions between claustrum and thalamus might be more developed in primates than in rodents. If confirmed, such differences would imply that rodents and primates might not share the same large-scale architectures for the regulation of cortical gain, salience, or precision. In rodents, thalamic influences on claustrum could operate predominantly in a unidirectional manner, whereas in primates reciprocal thalamo–claustral interactions might support a more elaborate coordination of distributed cortical processing. More generally, these considerations reinforce the need for direct comparative investigation of rodent and primate circuits, and caution against assuming that computational motifs inferred in rodents can be transferred unchanged to the primate brain.

## Abbreviations

AAV: adeno-associated virus
BU: bottom–up
EDR: exponential distance rule
fMRI: functional MRI
hdPC: hierarchical dendritic predictive coding
hPC: hierarchical predictive coding
NHPs: non-human primates
TD: top–down
VASO: vascular space occupancy
